# Reports on Cities, Towns, and Districts

**Published:** 1856-10

**Authors:** J. H. Houghton

**Affiliations:** Officer of Health.


### SANITARY AND SOCIAL SCIENCE.

### REPORTS OF CITIES, TOWNS, & DISTRICTS.

### REPORT ON THE SANITARY STATE OF THE TOWN OF DUDLEY.

By J. H. HOUGHTON, Esq., M.R.C.S., Officer of Health.

(*Continued from* p. 76.)

But, if the supply in those districts of the parish which are permeated by the company's pipes is so defective, what must be the condition of those two districts—St. John's and St. Andrew's—where there is not any artificial supply whatever? The following facts will suggest an answer to this question.

At a spot in St. Andrew's district where the population is most dense, the turnpike road divides into two, which diverge at an acute angle. At about two hundred or three hundred yards from the angle a street is cut across, which unites the two roads; the whole forming a triangular space, the base of which is from eighty to a hundred yards. On the two sides of this triangle, formed by the divergent roads, one hundred and forty-two houses stand, with the necessary privies, a number of pigsties, and one slaughter-house, one of the occupants being a butcher. From the back of these hundred and forty-two houses, there is a great fall on either side; and the refuse and drainage from the whole of them and their out-buildings gravitates to the centre, where a deep ditch has been dug to receive it, in some parts a yard or a yard and a half deep, and about the same width. This ditch is always filled with black mud, as thick as turtle-soup, which emits a most noisome smell when stirred. A number of small wells are dug along the side of this ditch, at distances varying from two to four feet; and the water from the ditch filters through the soil into them, and is used by many of the inhabitants of the houses for domestic use: and, indeed, so valuable is this water considered, that some of these pits *are bricked, and have lids with lock and key, to prevent the water from being stolen!*

Mrs. S., the wife of a small tradesman, residing in another part of St. Andrew's district, gave me and Mr. Thompson (member of the Local Board, who is engaged with me in endeavouring to remedy this state of things in St. Andrew's) the following account of the mode of obtaining water: "We carry all our clean water in buckets from the reservoir, and we are obliged to send for it every day; sometimes we send for it three or four times a day. I have often, when younger, carried *forty* buckets of water on my head in one week from the reservoir to my house." The reservoir belongs to the Canal Company, and is more than half a mile (over a very bad road) from that part of the hamlet in which Mrs. S. lives; so that she has often walked over forty miles a week (half of it with a heavy load on her head) in order to obtain this primary necessary of life. Her neighbours are all in the same condition.

There are a few pools in St. Andrew's, and the water from these is generally used for washing the houses. I have often seen two children standing at the pools together, one filling a bucket to carry water away, and the other washing a mop, on which most probably were the fæces of some child; and, on going into the houses whilst they are being washed with water so obtained, the stench is at times almost intolerable.

I have already alluded to the fact that a sub-committee of the Local Board (with Mr. Thompson, the active and intelligent manager of the British Iron Company's Works, near Dudley, for their chairman) is engaged in devising a plan for supplying St. Andrew's with water.

St. John's is a little better off for water than St. Andrew's, but not much. It derives its supply entirely from wells and from rain. The former are not drained by the mines so much as they are in St. Andrew's, in consequence of the hamlet being built on the basaltic rock.

In Dudley, we find a good illustration of the fact that towns built on hill-sides, without a sufficient drainage, are specially liable to the evils arising from bad sanitary arrangements. This is best illustrated in St. John's district, which is almost entirely built on the basaltic rock, which rises to a very great height above the level of the town of Dudley. From its geological formation, its great height, and the precipitancy of its surface, on a *primâ facie* consideration, we should think it must be free from bad sanitary influences. A little reflection, however, will show that this is not, and cannot be the case, whilst it is destitute of efficient drainage. In St. John's, the streets are laid out one above the other, in rows more or less parallel, each street being many feet above the one next below it. From this arrangement, two evils arise: one, that that side of the houses on the upper side is necessarily kept damp by the earth reaching a distance up the walls; the other, and the most important one, is, that the drainage from the upper houses, having no artificial course, naturally finds its way downwards, forming cesspools as it goes; and in many places, where the houses are built, as is frequently the case, with doors and windows only in front, these cesspools are formed by the houses themselves damming up the refuse.

My attention was first called to this fact, by an inquiry I was called upon to make into the cause of the prevalence of fever in a yard in St. John's district. On inspection, the yard was found to be one where one would least expect fever to be located. It was pleasantly situated, spacious, well paved and drained (as to the surface); the privies were better than usual, and better kept; there were no pigs; the houses were better built, and inhabited by a much more clean and careful class of people than we often find;—in fact, there did not appear to be any localising cause, and yet the place was full of fever. On inspecting the premises surrounding the yard, the cause was soon obvious. The drainage from the houses next above—from the privies (many overflowing) and the pigsties, had gravitated downwards, and formed a large cesspool at the back of the houses on one side of the court, and had flowed round a great part of the other side, thus almost surrounding the place with a pestilential atmosphere. This place is not unique in Dudley.

In consequence of the absence of drainage and plentiful supply of water, the use of water-closets in Dudley is quite the exception; the old privy with the cesspool being the rule, even in many of the best houses. In those parts of the town inhabited by the poor, the state of these places, previous to the formation of the Local Board, was incredible. In one place I found eight houses, with forty-seven inhabitants, with only one privy, and that in such a disgusting condition as to be totally unfit for use. In another, I found fifteen houses, with ninety-one inhabitants, and only two privies, and one of these had been *dedicated to the public*, and had become so foul and offensive, that the inhabitants refused to use it; so that, in fact, the *ninety-one people had only one amongst them*. One tidy-looking man told me that, in the eleven years he had lived in the yard, he had never entered a privy—"he always goes into the fields, which he considers more healthful." Before the "Board" was established, there was not any control over these nuisances; and, though the two cases mentioned are the worst I met with, there were many in the parish nearly as bad. The people have pretty well accommodated themselves to the circumstances under which they have been placed; the result being, that the back streets, courts, and other eligible places, are constantly found strewed with human excrements. The efficient remedy for this state of things will only be found by the compulsory use of water-closets, when the drainage is completed.

Next comes the "pig nuisance", which in Dudley is one of the most conspicuous of all pestilential nuisances, not only from the filth necessarily attendant upon keeping the animal under any circumstances, but specially from the number kept, and the filthy condition in which they are kept, as must be the case in any place destitute of water and drainage.

Numbers of facts might be adduced to prove the injurious effects of pigs in towns. I quote a few from my report to the Local Board on the progress of the cholera in 1854. On the 9th of October, 1854, three cases suddenly appeared at a place called "Primrose", which is a sort of long street in St. Andrew's district, built quite on an eminence, and in the country. Within fifteen yards of the back door of the house in which these persons lived, were *eight pigsties*, in the most offensive condition possible, and one of them close under the window where the poor creatures were lying. The fluid ordure from the animals escaped, ran about, and lodged in the inequalities of the surface, forming large bog-holes. On the 18th of September, the child of a shop-keeper in the High Street died. Mr. S. D. Fereday, in reporting the case to me, observed, "the sanitary state of the house is bad, *foul pigsties* surrounding it." On the 4th of November, a mother and her daughter were seized, at a place called "Sweet Turf", at about noon; before four the next morning, they were both dead. "At about ten yards from the front door of the house, which had no through ventilation, there are two foul pigsties; a large pit had been dug to catch the drainage from them; this had overflowed, and covered the surface for some fifteen or twenty yards square around it with the liquid pigs' manure." In another case, "a pigsty was under the bedroom window, and three others within two or three yards of the house"; and in another, "a lot of pigsties had been built against the gable end of the house, which in every other respect was placed in a good sanitary condition." Facts like these might be multiplied to a great extent.

Pig-keeping is a favourite "hobby" with the miners and iron-workers; and many pigs are kept in the most crowded and improper places. From the difficulties of keeping them clean at all, from the continued occupation of the owners, and, most of all, from ignorance or non-appreciation of the evils arising from the pigs, they are generally kept in the dirtiest possible condition; and, even when they are kept clean, they are only kept so by forming a manure-heap by the side of the sty, which, in a sanitary point of view, hardly lessens the evil.

The evil, however, is not at all confined to the working people; wealthy people and shopkeepers contributing their quota to the nuisance. Complaints have been made before the Local Board, by a medical man, who lives in one of the best houses, in the very best part of the town, of the nuisance arising from his neighbour's (an auctioneer and appraiser) pigs. The principal clerk, resident at the Banking House in the High Street, complained of one of his neighbour's pigs. Another surgeon made a similar complaint. And in one case a number of pigs were kept, by a wealthy individual, on a high manure-heap, placed against a wall which formed the back of some houses. The fluid from them had gradually soaked through the walls and ran down the inside of the houses, and positively drained into the oven where the people who lived in the yard had to bake their food.

The above and other facts having been pressed on the Local Board, they resolved to take the matter up. A sub-committee was formed, and accompanied me in an inspection of these and other evils of a similar nature; and so strong was their feeling, that they unanimously agreed to a report recommending the Board to prohibit pig-keeping altogether within the limits of the town; and a motion to this effect was carried at the Board.

The passing of the resolution was the signal for a great storm in the town. A public meeting was held, and resolutions condemning the measure of the Board passed; and a deputation waited upon the Board at its next meeting to present the resolutions. The "vested interests" and "liberty of the subject" were said to be interfered with; and finally, after an ample discussion into the whole matter, the question was referred to the General Board of Health, who decided that the Local Board had no power to enforce the resolution; and so the matter ended. The pig-keepers triumphed; the pigs got a reprieve, and continue to "wallow in their mire", undermining the health, and destroying the comfort and happiness of very many of the people.

At the same time, a similar scene was enacted in Leicester, with a similar result; and it seems that the nuisance can only be dealt with in detail, which is tantamount to saying that it cannot be dealt with at all; for, from various motives, it is very seldom found that one neighbour will complain of another; and out of numbers of cases, in which I have been requested privately to remove certain pigs, I have found but very few in which complainants were willing to appear in the matter: they would rather submit to the nuisance from the pigs, than to the nuisance of complaining, and so creating ill-feelings. Thus this monster evil remains without a remedy; and it will remain, unless provisions for its abolition are enacted by parliament.

The slaughter-houses have been a source of much disease in Dudley; but since the Board has been established, they have been regulated by judicious bye-laws, and closely inspected, and the evils arising from them have been very much mitigated. Still, it is almost impossible for slaughter-houses to exist in towns, without injury to the health of the people; and it is to be hoped that they will some day be entirely prohibited, and proper accommodation for the purpose provided in suitable situations.

The lodging-houses are all under inspection, and generally speaking are well conducted and cleanly.

The figures before given will show that the proportion of houses to the people is ample to prevent overcrowding; but the distribution is unequal, and in some cases it does exist, and is the cause of disease. During the cholera, cases were observed where no other obvious cause presented itself; and I have frequently seen it cause fevers in various degrees, and have also found the fevers where overcrowding has existed singularly obstinate and difficult to treat. Amongst all sanitary evils, there is none so difficult to deal with as overcrowding; for it is generally caused by circumstances over which the persons subjected to it have no control, and which they cannot alter, and which no law can reach—I mean the pecuniary resources of the people.

Such is a brief view of the sanitary condition of Dudley; and I fear it applies pretty much to the towns of the South Staffordshire Coal Field. The effects of this sanitary state are stamped on the people. Cleanliness, as a general rule, is rare. The occupations of the people are of necessity dirty; and the deficiency of water, and familiarity with local filth arising from deficient drainage, soon obliterate all habits of cleanliness, except in particular cases. I have in many cases watched families, who have come from the agricultural districts to seek employment, with habits of the most scrupulous neatness, gradually sink into those of the greatest inattention to personal and domestic cleanliness, as they admit, from the very impossibility of keeping themselves otherwise.

The Public Health Act has already been applied to some of these towns, and measures are being taken to secure its application to others. It is to be hoped that it will be extended to all; and that thus, in the course of a few years, an improved sanitary condition of its thousands may be attained, and that on this foundation a grand superstructure may be raised—the elevation of the moral, the social, and the religious status of the people.

---

